# Effect of Road Safety Education on Road Risky Behaviors of Spanish Children and Adolescents: Findings from a National Study

**DOI:** 10.3390/ijerph15122828

**Published:** 2018-12-12

**Authors:** Francisco Alonso, Cristina Esteban, Sergio Useche, Natura Colomer

**Affiliations:** DATS (Development and Advising in Traffic Safety) Research Group, INTRAS (Research Institute on Traffic and Road Safety), University of Valencia, 46022 València, Spain; datspublications@gmail.com or francisco.alonso@uv.es (F.A.); cristina.esteban@uv.es (C.E.); natura.colomer@uv.es (N.C.)

**Keywords:** road safety education, RSE, children, adolescents, risky road behaviors, road safety, traffic crashes

## Abstract

Background: Road Safety Education (RSE) is widely known as a reliable determinant of the future results for what concerns health and welfare and as an undisputable factor which contributes to the social behavior of individuals and to their mid- and long-term road safety outcomes. However, its development has been relatively scarce in most countries, a fact which has contributed to letting matters as delicate as traffic crashes, largely explained by road misbehaviors, continue to be a prevalent problem, thus affecting the health of the community. Objective: The aim of this study was to describe the relationship between demographic and RSE-related variables and the self-reported road risky behavior of Spanish students. Methods: For this cross-sectional study, a representative sample of 4062 (51.5% males and 48.5% females) participants attending primary (47.5%), secondary (40.7%), and high school (11.7%) was gathered through a national survey on RSE and road behaviors. Results: A set of significant associations between demographic factors, RSE variables, and self-reported road behaviors was found. Furthermore, Structural Equation Modelling (SEM) allowed us to establish that age and observed misbehaviors (positively), and attitudes towards road safety and risk perception (negatively), have a direct link with the road risky behavior of children and young people. The knowledge of traffic rules was not a significant predictor of road behavior. Conclusions: The results of this study show that, together with demographic factors such as age, RSE-related variables have an effect on the road behavior of children and young people. They also suggest the need for strengthening actions to be implemented in road safety (Road Safety Education) at scholar and community levels.

## 1. Introduction

Road crashes are recognized as one of the worldwide leading causes of mortality among young people. They account for approximately 35–40% of injury-related mortalities among teenagers and young adults in Western countries, and risky road behaviors are one of their most important predictors [1,2]. Globally speaking, pedestrians constitute the largest category of children involved in road traffic crashes. In high-income countries, between 5% and 10% of children suffering road traffic injuries are pedestrians, while in low-income and middle-income countries, the proportion ranges from 30% to 40% [3]. This number is appallingly rising, and children injured or killed while traveling as passengers in cars are a serious concern for high-income countries; such cases can account for up to 50% of children’s traffic deaths. Moreover, half of the estimated 1.2 million fatalities worldwide occurring every year involve vulnerable road users (VRUs) who are killed in road crashes, with children and elderly people being overrepresented among the victims. In parallel, 50 million people are injured and live with long-term adverse health consequences [4,5].

### 1.1. Perception of Road Risk and Behavioral Education through RSE

It is clear that children perceive neither traffic signals and norms nor the overall reality in the same way as adults do. Moreover, the pedestrian safety of children is likely influenced by individual differences in temperament and personality [6,7], and also by the observed behaviors in, e.g., parents or relatives, professors, peers, and other significant members of their microsocial system [6,8]. That is why a child’s development must be considered in order to introduce the best educative interventions, since it has been shown that Road Safety Education (RSE) tends to be more effective when started at a young age. The environmental risks, such as the location of schools and recreational or play areas, are also relevant for the safety of pedestrian children since they are more likely to be hurt near schools [8]. Thus, behavioral approaches and traffic safety education without any modification of the traffic environment might not effectively prevent the occurrence of pedestrian injury in low and middle-income countries with poor traffic conditions [2,9].

Regarding gender differences, it is known that females reported significantly higher perceived risk towards unsafe driving than males [10,11], and, in related works, it seems to emerge that people are more easily sensitized to risk than to safety and that strengthening risk perception through systematic interventions (such as the ones used in RSE-related paradigms) may result in positive behavioral changes [12]. For all these reasons, affective and motivational mechanisms, including attitudes and perceptions, must be taken into account when developing road safety educative interventions.

### 1.2. Road Safety Knowledge and Behavioral Factors

Global road safety knowledge is an independent protective factor for road traffic injuries. Students with a high level of risky traffic behaviors or with low knowledge of road safety rules are more likely to suffer road traffic injuries [13].

In previous applications of some RSE interventions, well-structured and systematic programs have been proven to have an impact on some protective results of socio-cognitive and behavioral factors, although the results have been shown to be quite dependent on the beneficiaries’ profiles [14]. This shows the importance that programs of comprehensive intervention have (including road safety knowledge and education, strategies of behavioral change, and management of environmental risk), and therefore they must be rightly and properly planned.

Much of the literature on the safety of pedestrian children discusses the importance of exposure to traffic and of the acquisition of skills in real-life traffic environments [15], particularly of developing an awareness of traffic and learning fundamental road safety practices initially under adult supervision and finally leading to independent journeys. In addition, although it is recommended to keep children away from dangerous road traffic scenarios, the evidence has shown that road safety education must involve their performance in realistic situations as much as possible in order to provide them with experiential tools for strengthening ulterior positive road behaviors [16].

### 1.3. Attitudes towards Road Safety and Road Risky Behaviors

Recent empirical evidence has shown that road-risky behaviors present relevant particularities according to demographic variables, such as age or gender [11]. For example, young males are more prone to presenting negative attitudes towards traffic rules than females, who are less involved in alcohol-related and speeding-related crashes than males [17]. However, Cordellieri et al. [18] showed interesting results regarding gender differences which suggested that both males and females do not have the same risk perception regarding potentially hazardous situations on the road; females showed, overall, a higher road-risk perception rate. Therefore, this difference between risk perception and concern could explain some differences in the reduction of hazardous behaviors and in the frequency of road causalities.

In parallel, the attitude of drivers translates knowledge into action and it is one of the key factors contributing to driving behavior [19]. Other evidence confirms that individuals with a high propensity for driving behaviors associated with an increased risk of road traffic crashes are more likely to have negative attitudes towards traffic safety, and therefore attitudes are correlated to reported behaviors [20]. When the behavior is not completely planned, complementary factors such as self-efficacy are especially important for explaining the observed actions of road users [21]. Furthermore, both attitudes and perceived risk have been shown to reliably predict unsafe driving behavior and involvement [22,23,24,25].

### 1.4. Road Safety Behavior in Children and Adolescents

According to Gårder [26], the use of engineering combined with education and institutional enforcement in road behavior programs is the most effective strategy for improving the safety of pedestrians. Based on previous findings, pedestrian behaviors can be explained through different theories such as, for instance, the self-determination theory, which highlights the importance of intrinsic motivations in behavioral self-regulation [27]. The Protection Motivation Theory, which focuses on risk perception, highlights the importance of perceived severity (i.e., the degree of harm that could possibly be derived from taking the risk) and perceived vulnerability [28].

Behavioral intention is a key predictor of behavior [29], and external social norms (i.e., descriptive and injunctive norms) as well as internal norms (i.e., personal norms) are important if the objective is to generate voluntary safe behaviors [30]. Moreover, the motivation behind an intentional risky driving behavior is determined by the attitude towards that behavior, the subjective norm, and the perceived control over one’s own behavior.

Based on the findings from the research of Guggenheim and Taubman-Ben-Ari [31], educational programs in RSE aimed at adolescents should promote behaviors that take advantage of the potentially positive influence of friends in order for young drivers to be encouraged to take more responsibility when driving with friends. In this sense, the findings from one study supports that social norms may influence the speeding behavior of teenagers, and this relationship may operate through perceived risk, suggesting an important effect of the influence of friends on teenage drivers [32]. Accordingly, it is necessary to focus the research on the phenomenon of influence among peers on what concerns safe behaviors on the road.

Finally, scientific evidence has shown that RSE may have some positive effects if good practices are adopted, such as the programming of pedagogical objectives, the verification of the trainers’ competences, the adaptation of methods in order to achieve the purposes, and the testing of the effects produced by the impact [1]. Far from being considered just a mere school subject, RSE must be part of a lifelong learning process. 

Curiously, according to Thompson [33], the major challenges in educative interventions do not consist of focusing the attention on the merely observed behavior but, rather, of fostering good practices such as the use of interactive learning methods, the enhancement of positive attitudes towards road safety, the development of better social competences, the integration of volunteer trainers into programs, and the direction of efforts towards the use of realistic training scenarios. Of course, this implies a major integration of the educational system and all its involved stakeholders [34].

### 1.5. Study Framework

The most relevant theoretical bases of this study are the relationships between road safety education (included in the literature and retrieved across an extensive bibliographic review) and its later outcomes in terms of traffic safety. Both factors are complex and difficult to assess, keeping in mind the large number of variables they involve. However, recent scientific evidence has demonstrated an existing relationship between road safety education and different key variables, such as attitudes towards road safety, risk perception, observed and reported behaviors on the road, subjective well-being, and health outcomes. According to this approach, road safety and health should be treated from a comprehensive perspective, i.e., with consideration for people’s biological, psychological, and social aspects, or involving some key stakeholders such as parents, members of the educational system, and other institutions. Moreover, it is important to understand the factors associated with the learning of road safety in order to prevent future risky behaviors, traffic crashes, and, also important, to promote awareness and risk perception among children. Therefore, this article was framed within a large-scale project of research on road safety, developed by the University Research Institute on Traffic and Road Safety (INTRAS). This global research on road safety education and environmental issues of children used a questionnaire composed of a set of items divided into different sections. The questionnaire was used to collect socio-demographic and psychosocial data from participants and their parents. The study described in this article is based on some items from the section titled “*Road safety education in children and young people*”.

### 1.6. Objectives and Hypotheses

This study had two main objectives: First, to describe the relationship between age, observed road behaviors of parents and peers, road safety education-related variables, and the participants’ self-reported risky behaviors; and second, to assess the effect of these variables on the participants’ road behavior through a path analysis.

Regarding our hypotheses, the expected results of this study, according to each objective, were first, that age, observed road behaviors, and RSE-related variables will present significant associations; and second, that age, observed behavior, and RSE variables would have an effect on the explanation of the participants’ self-reported risky behaviors on the road.

## 2. Materials and Methods 

### 2.1. Sample

For this cross-sectional study, a total sample of n = 4062 Spanish students (2092 males, being 51.5% of the sample, and 1970 females, representing 48.5% of the sample) was used, all coming from 19 different provinces of Spain. The mean age of the full sample was $\bar{x}$ = 12.46 ($\bar{x}$ = 12.56 male and $\bar{x}$ = 12.37 female); SD = 3.01 years. 47.5% of participants were primary school students ($\bar{x}$ = 10.04 [$\bar{x}$ = 10.14 male and $\bar{x}$ = 9.96 female]; SD = 1.57 years of age); 40.7% were in secondary school ($\bar{x}$ = 13.91 [$\bar{x}$ = 13.87 male and $\bar{x}$ = 13.95 female]; SD = 1.48 years of age), and 11.7% of them were attending high school or professional training degrees ($\bar{x}$ = 17.19 [$\bar{x}$ = 17.27 male and $\bar{x}$ = 16.99 female]; SD = 2.39 years of age).

### 2.2. Study Design and Procedure

Participants were invited to participate in the study through the distribution of a national survey on Road Safety Education in which children answered a set of questions on this topic in the classroom. The global response rate (completed and totally answered questionnaires) was approximately 97%, from a total of 4200 students initially asked to partake, representing a fairly high participation rate. The sample size was established according to the calculation of statistical representativeness carried out using the Raosoft^®^ sample size calculator, based on the total population and on the estimated sample needed to fulfill the basic parameters.

The sample was obtained from schools which had previously agreed to cooperate with the research project, and surveys were applied in the classroom with the authorization and cooperation of the educational staff (teachers, directors, and coordinators) involved in the pedagogical work of the center. Regarding the sampling technique, for this study, we employed a convenience (non-probabilistic) sampling grounded on the accessibility to the population and on their willingness to participate (or not) in the study. Bearing in mind the mean age of participants and the need for ensuring an adequate understanding of the questions raised, (a) the instruments were preliminarily assessed in a pilot study in which any hard-to-understand questions or infrequent terms were amended; (b) a member of the research staff was always available to advise them in person during the completion of the questionnaire; and (c) the survey was always conducted with explicit guarantees for the anonymity of its participants, emphasizing the existing laws on data protection and the fact that the information would only be used for statistical and research purposes in order to minimize the possibility of finding biases such as subjective reporting, social desirability, or acquiescent responses. We also kept in mind that most of participants were under aged, which is a reason why a previous permission signed by the school institutions and by the associations of parents had to be elaborated and agreed upon. All participants were initially informed about the importance of answering honestly to all the questions, as well as about the non-existence of wrong or right answers.

### 2.3. Instruments

For this study, a paper-based questionnaire composed of four sections was elaborated: First, a short summary of demographic data (i.e., age, gender, city/region of residence, current educational level) was completed in order for the researchers to characterize participants.

A second section was used to assess the participation of students in Road Safety Education activities and all their related factors, such as the type of interventions, their duration/intensity, the value attributed to them, and the scenarios employed for these interventions.

A third section was designed to assess the participants’ perception of several factors related to road safety education using the following sections.

Firstly, for assessing the knowledge of traffic rules (*α* = 0.70) and the ability to identify traffic signals (*α* = 0.68), a 12-item scale (6 for each factor) was used; it presented a series of statements to be answered as false or true in order to determine the participants’ knowledge of basic traffic norms and signals (example item: Rear seat passengers in a vehicle are NOT required to wear a seatbelt). Attitudes towards road safety were assessed using a 6-item scale (*α* = 0.73) that presented a series of statements related to safe and unsafe attitudes of participants as road users (example item: Even if using the seatbelt were not mandatory, I would still use this safety element). Thirdly, risk perception was measured using a 12-item scale (*α* = 0.58) that presented some potentially risky road situations and asks the respondent to state the degree of risk implied in these situations using a Likert scale and answering the question, “How much risk you perceive in the following situations?” (example item: Using the cellphone while walking), based on the following perceived risk levels: 0 = None (“*It does not constitute any risk for me; I do not think this could lead to an accident*”); 1 = Medium risk (“Although it might not necessarily cause an accident, it is true that this could put me in danger”); and 2 = Very high risk (“It is definitely very dangerous and would surely put me at risk of suffering an accident”). 

Finally, the fourth section was composed of two sets of items: For the first about self-reported risky behaviors, a 6-item questionnaire (*α* = 0.82) was used for asking whether the participants usually performed (or did not perform) some risky behaviors (example item: If I am about to cross the road and the pedestrian traffic light has started to blink, I cross running, as it will almost immediately change to red). As for the second, a 12-item frequency scale (ranging from 0 = Never to 2 = Too Often) was used to ask them how often they observed risky road behaviors in their parents and peers (6 items for parents and 6 for peers) (example item: How often do your parents drive after drinking alcohol?).

The average time needed for filling out the survey in a pilot application (data not included in the final sample) with n = 50 Spanish children and young students was $\bar{x}$ = 13.5 min.

### 2.4. Statistical Analysis (Data Processing)

Firstly, the raw database was checked and transformed into numerical data, and study variables were calculated. For the case of demographics, data was coded and labelled, and, for the case of sub-scales, the respective items of each were summed using their scoring guidelines; negative items were reversed in order to obtain harmonized global scores on the study factors. Although only few questionnaires contained missing data and the sample was extensive, missing values were transformed through data imputation by regression coefficients (procedure available in AMOS software) in order to respect the distribution of the data and their measurements of central tendency.

After performing basic descriptive analyses, a bivariate correlation (Pearson) analysis was performed to establish potential relationships among the study variables in the case of this sample of Spanish students. Furthermore, the associations between demographic data (age), RSE-related variables, observed road behaviors, and self-reported road risky behaviors were tested using path analysis (Structural Equation Modelling [SEM] with maximum likelihood estimations) with the following significance parameters: *p* < 0.05, *p* < 0.01, and *p* < 0.001. All statistical analyses were performed using ©IBM SPSS (Statistical Package for Social Sciences), version 24.0 (Armonk, NY, USA, 2016), and ©IBM SPSS AMOS, version 22.0 (Armonk, NY, USA, 2015), principally used for conducting structural analyses.

### 2.5. Ethics

In order for this study to be conducted, the Research Ethics Committee for Social Science in Health of the University Research Institute on Traffic and Road Safety at the University of Valencia was consulted, certifying that the research subject to analysis responded to the general ethical principles, currently relevant to research in Social Sciences, and issued a favorable opinion to be carried out in Spain. Furthermore, an informed consent statement containing ethical principles and data treatment details was used, and it was signed by participants before answering the questionnaire.

## 3. Results

### 3.1. Descriptive Findings

The bivariate correlation analysis (see Table 1) allowed us to establish significant measures of association among study variables related to the Road Safety Education of Spanish students. Firstly, age was negatively and significantly associated with positive attitudes towards road safety and risk perception (the higher the age, the lower the risk perception, and the less favorable attitudes observed). On the other hand, age was positively associated with the knowledge of both traffic signals and written traffic norms.

Regarding some important correlations found directly among RSE-related variables, it was found that positive attitudes towards road safety were significantly associated with the amount of misbehaviors on the road observed in parents and peers [−], the identification of traffic signals [+], the risk perception [+], and the risky behaviors on the road [−]. Furthermore, risk perception was significantly associated with the amount of road misbehaviors observed [−], and with the knowledge of traffic norms [+]. Finally, risky behaviors on the road were positively [+] correlated to age and to observed road misbehaviors (in both parents and peers), and negatively [−] correlated to road safety attitudes, as shown in Table 1.

### 3.2. Explaining Road Risky Behaviors: SEM Modelling

Based on the theoretical roots presented in the introduction, the effect of variables related to Road Safety Education on self-reported risky road behaviors of Spanish students was examined using a SEM (Structural Equation Modeling) approach. Using the SPSS AMOS path analyses, the hypothesized structural model was adjusted in order to fit the data while considering the parameters of the full sample of n = 4062 participants, accomplished with the minimum sample size suggested by the literature [35].

**Figure 1:**
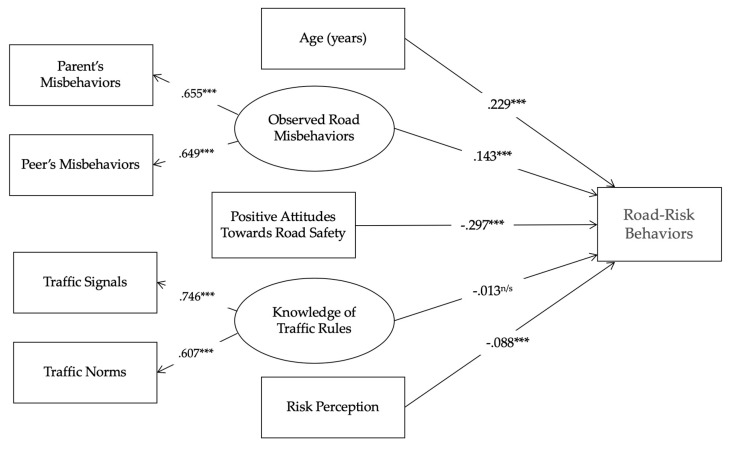
Path structural model showing standardized path coefficients for self-rated Risky Behaviors on the Road: * *p* < 0.05; ** *p* < 0.01; *** *p* < 0.001.

A baseline (a priori) model did not fit the data well (*x*^2^_(20)_ = 1226.50, *p* < 0.001; Normed Fit Index (NFI) = 0.725; Comparative Fit Index (CFI) = 0.724; Root Mean Square Error of Approximation (RMSEA) = 0.108) and needed to be adjusted. Therefore, several modifications were made. Firstly, non-significant and very low paths were set to zero. Secondly, a very large Modification Index that pointed out a relevant relationships between the independent variables and risky behaviors was used. These modifications made the model even more parsimonious, and the model fit that resulted was adequate. The resulting Structural Equation Model, more parsimonious and reporting better fit coefficients (*x*^2^_(18)_ = 10.213, *p* < 0.05; NFI = 0.979; CFI = 0.984; RMSEA = 0.039; Minimum Discrepancy/Degrees of Freedom (CMIN/DF) = 0.57), all of them acceptable and indicating a good model fit [36,37], is presented in Figure 1.

In short, the standardized path coefficients (see Table 2 and values next to solid lines in Figure 1) of the model show positive associations between age (*β* = 0.229 ***), observed road misbehaviors (*β* = 0.143 ***), and risky behaviors on the road (dependent variable). Put another way, individuals with greater age and observing more road-risk behaviors in their parents and peers also tend to present more self-reported road misbehaviors.

On the other hand, negative relationships were found between risk perception (*β* = −0.088 ***), positive attitudes towards road safety (*β* = −0.297 ***), and risky behaviors. In other words, the greater the scores in risk perception and positive attitudes towards road safety, the lesser is the score in risky road behaviors reported by children and adolescents. Nevertheless, the self-reported level of knowledge of traffic rules computed through the knowledge of traffic signals and written norms did not present an explanatory role in the self-reported road behavior of participants (*β* = −0.013 ^N/S^), bearing in mind that the statistical relationship was not significant.

## 4. Discussion

This study had two main objectives: First, to describe the relationship between age, road behaviors observed in the social environment and variables related to road safety education in Spanish young people and children, as well as their risky road behaviors; and second, to assess the effect of these variables on the participants’ self-reported risky behavior on the road using a path analysis. One key aspect of this research was our approach, in which we considered the road safety of children and young people not only as a whole, but as a set of components which mutually influenced each other and, as the results confirmed, can influence the road risky behavior of participants, as also described by different studies [15,16,38]. 

Regarding the first objective of the study, we found a set of significant associations between demographic factors, road behaviors, and issues related to road safety education. Bearing in mind the age range of the sample (and that some of these elements work differentially for adult road users), we will discuss the results within the frame of the evidence related to this age group. First, a relevant set of positive associations between knowledge of traffic rules, positive attitudes towards road safety, and risk perception suggests the need for strengthening these elements in road safety education interventions, especially when considering their proven association to further risky/safe road behaviors [19]. Furthermore, the observed road behaviors had a correlation with the self-reported risky behaviors of participants in accordance with some studies supporting the influence of observed road behavior on the one performed by children and young road users [32,39,40]. However, we must face the rise of two difficult issues regarding this point: First, that we used a self-report measure for risky road behaviors (see Section 4.1), and second, that the observed behaviors constitute an aspect that is complementary to RSE in the acquisition of safe road habits, but at the same time, it does not fulfill the need for exerting major efforts in improving safe attitudes and risk perception of road users, considering that those factors also have an effect on the own road behavior [33,34]. Finally, it is also worth discussing the non-significant bivariate association between knowledge of traffic rules and signals, road risk perception, and self-reported behaviors on the road; this supports the idea that, although rule knowledge and risky road behavior do not present a direct link, when modelled together with other variables related to road safety education (i.e., age and observed behavior), they make it possible to explain the behavioral outcomes of children and young people. This is an essential part of the second objective of the study.

Subsequently, for the Structural Equation Model obtained with good fit and significant paths in accordance with the theory, we will discuss the model’s principal components and explain the paths, basing our reasoning on the study variables. The first variable included in the path model was age. In this regard, former studies have already confirmed that age is usually related to a decreasing trend in the performance of risky behaviors when groups of young and adult road users are analyzed together [34,40,41]. Nevertheless, it is necessary to highlight that in our research the oldest age of the participants corresponded, overall, to teenagers for which we observed a rebound of the hazardous behavior. Therefore, in our case, risky behaviors are more common among adolescent and young pedestrians [42]. Following the same line, there are no protective factors against the commission of risky traffic behaviors related to the older population of our study.

Overall, the findings of our research show that the road misbehaviors observed by participants (i.e., Spanish children and young students) influenced their hazardous behaviors. Some previous analyses have reached the conclusion that the individual road behaviors that need to be addressed involve, apart from children, some key stakeholders, such as their parents and teachers [43] because parents may influence their children’s road behavior in different ways. Following this line, as an example, cautious drivers are more likely to have prudent children [44], a fact that could highlight the importance of the imitation of positive attitudes in safety behaviors. Still on the subject, according to Taubman-Ben-Ari et al. [39], the risky behaviors performed by parents are usually repeated by their children. Beyond this fact, it seems clear that psychological factors such as risk perception, the behavior of parents, and their attitudes towards traffic safety may affect children’s road safety outcomes, which is one of the key findings of the study on the role of parenting in road safety behaviors of children and adolescents [45].

Another major variable influencing road behavior is the promotion of knowledge and understanding of traffic rules and situations, which is one of the pillars of RSE [1,34]. As expected, in our study, those children who displayed a lack of knowledge of traffic signals and norms tended to take higher risks in their road behaviors. However, it is also remarkable what other studies have proven, which is the fact that an increase in the knowledge of road safety does not necessarily translate into improved behavior in real traffic situations [46].

Following this point, the literature has progressively shown that attitudes are strong predictors of pedestrian behaviors as well [41] and that performing hazardous behaviors is negatively correlated with positive attitudes towards road safety [34]. Thus, when we analyze the relationship between these two variables, it seems that positive attitudes towards road safety decreases risky behavior, and the results of our study reinforce this statement.

Although several studies on the link between safe behavior and attitudes towards traffic safety issues have been conducted, especially among young drivers [47,48], there are no standard similarities nor general possible predictions for the attitudes of young pedestrians. Consequently, this highlights the importance of identifying children who are particularly prone to adopting risky and potentially harmful road behaviors [49]. Besides, attitudes and cultures are related to the risky behaviors of pedestrians. Such is the case of the vertical collectivism approach (which defines the self in relation to others with an emphasis on conforming to authority and hierarchy), which was found to be associated with safe pedestrian attitudes among young people [50]. First and foremost, it is essential to explore the implication of the associations of cultural factors in traffic safety educational programs.

Regarding the last point, the results found in a study which addressed a similar research topic showed that the average score in self-reported road risky behaviors was significantly higher in the case of participants with less risk perception [11,34]. This outcome is consistent with the negative and significant association between these two variables that we found both in our study and other empirical experiences in the field of safe road behaviors [51]. In this regard, other findings show that, for instance, when young people are simultaneously using their cellphones and crossing the street, this influences their road behavior, which generally becomes considerably riskier [52]. These new patterns of street crossing combined with cellphone use are endangering the road behavior of children and adolescents with non-desirable distractions that compromise an accurate risk perception and represent, of course, the need for involving new potentially positive and hazardous elements present in the road environment. It is also worth remarking the need for implementing interventions on RSE from a young age, bearing in mind both our findings, which show that younger participants receiving RSE inputs also report better behavioral outcomes, and that of different studies showing that the efficacy of road safety-related skills could be maximized through an early and systematic intervention [6,53]. It requires, of course, a major articulation with the educational system, policymaking, and its related stakeholders.

These issues should also become active values for the design and application of road safety education strategies, considering key elements such as age, road safety skills, and the needs of children and young people, and the proven influence of RSE on their future behavioral outcomes as road users.

### 4.1. Limitations of the Study

Although the size of the sample was considerably large and the main statistical parameters needed were overall accurately and satisfactorily tested, some factors related to the design, data collection, and scope of the results should be listed as potential sources of bias in this study. First of all, this study followed a self-report method, thus enhancing the potential occurrence of common method biases as well as potential biases in the responses provided by participants [54]. In short, and specifically if we bear in mind the age groups addressed by this research, although we put special effort into guaranteeing anonymity and highlighting the importance of obtaining sincere answers, phenomenon such as social desirability and acquiescence could be still elicited together with potential bias that may involve young people and children participating in self-report studies within the academic context, as already listed in some previous empirical studies [55,56]. In this sense, even just the fact that the research was conducted in the classroom may have involved a certain predisposition of the participants to provide “positive” or “desirable” answers in order to please the researchers, even though the “non-existence of wrong or right responses” had been emphasized during the introductory phase of the questionnaire. In other words, the single fact of performing school-based research may exert a certain biasing effect on some of the study subjects, which has to be minimized through an adequate reduction and analysis of the data.

Furthermore, there were some sources of bias related to the complexity of the questions and to the tasks proposed to our participants. Although the questionnaire was initially tested through a pilot phase showing positive results in terms of comprehensibility and clarity and amending potentially challenging terms and difficult questions, which were appropriate for the age range involved in the study (from primary to high school), some basic processes were still more comprehensible for older students, such as in the case of reading/writing tasks and some terminology that, although simple, could be better understood by the older groups of students involved in the study. Regarding the sample distribution, although every school cycle had a considerably large number of participants, the smallest group (high school) was composed of 475 individuals, a relatively small number compared to the other two. In this sense, some further statistical comparisons or in-depth analyses could be limited by the disproportionality among the study’s sub-groups. Bearing in mind the need for applying not-too-extensive questionnaires to the participants, we also did not use crosscheck questions, though we would like to raise the suggestion of using them as a control measure for biased responses provided by respondents for further studies in the field.

Finally, it is worthy suggesting that RSE-related factors could be also strengthened outside the school system. Other scenarios, such as the mass-media and institutional campaigns, also offer behavioral improvements for different road users, including the young population. For instance, evidence has shown that road safety campaigns coincide with a 10% reduction of crashes, especially those which involve personal communication, billboards and social media-related strategies to deliver their message [57,58]. A deep exploration of different factors, both at an individual level and within the social context, through qualitative data collection, could also be particularly useful and important, since it could facilitate the study of the subjects’ perceptions and opinions on road safety-issues, a factor that may maximize the development of more inclusive and effective policies in this regard.

## 5. Conclusions

The results of our study show that (once our theoretical considerations were tested through path analysis) the variables approached by Road Safety Education programs have a statistical influence on the risky behaviors reported by children and adolescents. It also suggests the necessity for producing and strengthening actions to be implemented in RSE-related interventions, taking into account all the above-mentioned elements and the need for articulating it with the educational system. 

In other words, the results shown in this study allow us to conclude that, together with demographic factors such as age, RSE-related variables have an effect on the road behavior of children and young people. In this sense, behaviorally-based emphasis on interventions related to road safety education may improve the children’s’ future road behavioral outcomes and, thus, their pedestrian safety.

Finally, it is worth stating that this paper may contribute to the understanding of RSE-related factors influencing the road behavior of children and adolescents, bearing in mind the current scarcity of empirical studies in this regard. This study also shows the importance of the involvement of the educational system and its related stakeholders in the labor of strengthening road safety skills of individuals since the early stages of life.

## Figures and Tables

**Table 1:** Bivariate correlations among study variables.

Study Variable		2	3	4	5	6	7	8
**1**	Age (years)	0.090 **	0.316 **	−0.071 **	0.041 *	0.120 **	−0.087 **	0.286 **
**2**	Observed Road Misbehaviors (Parents)	1	0.176 **	−0.256 **	−0.025	−0.119 **	−0.179 **	0.203 **
**3**	Observed Road Misbehaviors (Peers)		1	−0.195 **	0.065 **	0.026	−0.109 **	0.250 **
**4**	Positive Attitudes Towards Road Safety			1	0.025	0.165 **	0.357 **	−0.344 **
**5**	Knowledge of Traffic Signals				1	0.083 **	0.008	0.011
**6**	Knowledge of Traffic Norms					1	0.139 **	−0.057
**7**	Risk Perception						1	−0.217
**8**	Road Risk Behaviors							1

** Correlation is significant at 0.01 level (2-tailed). * Correlation is significant at 0.05 level (2-tailed).

**Table 2:** Frequency and percentage of each gender and each age group with or without anxiety symptoms.

Dependent Variable		Independent Variable		Estimate ^1^	S.E. ^2^	Std. Estimate ^3^	C.R. ^4^	*p*
Risky Road Behaviors	<---	Age		0.100	0.009	0.229	−11.074	***
Risky Road Behaviors	<---	Risk Perception		−0.045	0.011	−0.088	−4.159	***
Risky Road Behaviors	<---	Observed Risky Behaviors		0.114	0.017	0.143	6.656	***
Risky Road Behaviors	<---	Knowledge of Traffic Rules		−0.012	0.020	−0.013	−0.580	0.562
Risky Road Behaviors	<---	Positive Attitudes towards Road Safety		−0.286	0.020	−0.297	−14.336	***

^1^ SPC = Estimated Path Coefficients (can be interpreted as linear regression weights). ^2^ S.E. = Standard Error. ^3^ Standardized Path Coefficients. ^4^ C.R. = Critical Ratio. *** Significant at level 0.001; ** Significant at level 0.01; * Significant at level 0.05.
